# Bioaerosol Sampling for Respiratory Viruses in Singapore’s Mass Rapid Transit Network

**DOI:** 10.1038/s41598-018-35896-1

**Published:** 2018-11-30

**Authors:** Kristen K. Coleman, Tham T. Nguyen, Su Yadana, Christophe Hansen-Estruch, William G. Lindsley, Gregory C. Gray

**Affiliations:** 1 Emerging Infectious Diseases Programme, Duke-NUS Medical School, Singapore, Singapore; 2 Duke University School of Medicine, Durham, USA; 3 Health Effects Laboratory Division, National Institute for Occupational Safety and Health, Morgantown, West Virginia USA; 4 Division of Infectious Diseases, School of Medicine and Global Health Institute, Duke University, Durham, North Carolina USA; 5 Global Health Research Center, Duke Kunshan University, Kunshan, China

## Abstract

As a leading global city with a high population density, Singapore is at risk for the introduction of novel biological threats. This risk has been recently reinforced by human epidemics in Singapore of SARS coronavirus, 2009 pandemic H1N1 influenza A virus, and enterovirus 71. Other major threats to Singapore include MERS-coronavirus and various avian and swine influenza viruses. The ability to quickly identify and robustly track such threats to initiate an early emergency response remains a significant challenge. In an effort to enhance respiratory virus surveillance in Singapore, our team conducted a pilot study employing a noninvasive bioaerosol sampling method to detect respiratory viruses in Singapore’s Mass Rapid Transit (MRT) network. Over a period of 52 weeks, 89 aerosol samples were collected during peak MRT ridership hours. Nine (10%) tested positive for adenovirus, four (4.5%) tested positive for respiratory syncytial virus type A, and one (1%) tested positive for influenza A virus using real-time RT-PCR/PCR. To our knowledge, this is the first time molecular evidence for any infectious respiratory agent has been collected from Singapore’s MRT. Our pilot study data support the possibility of employing bioaerosol samplers in crowded public spaces to noninvasively monitor for respiratory viruses circulating in communities.

## Introduction

A high population density, international tourism and trade traffic puts Singapore at a high risk of novel emerging respiratory epidemics. This risk has been recently reinforced by human epidemics in Singapore of severe acute respiratory syndrome-associated coronavirus (SARS-CoV)^1^, 2009 pandemic H1N1 influenza A virus^2^, and enterovirus 71^3,4^ (which causes hand, foot and mouth disease). Other major threats to Singapore include Middle East respiratory syndrome-related coronavirus (MERS-CoV)^5,6^ and various avian^7^ (H7N4, H7N9, H5N2, H5N1, etc.) and swine^8^ (H3N2, H1N1, H1N2) influenza A viruses, especially if they become highly transmissible between humans. For instance, the first human case of A(H7N4) was confirmed in February 2018^9^, and a total of 1,625 human cases of A(H7N9) have been reported since 2013^10^, with 47% (766) in the 5^th^ wave of the epidemic from October 2016 to September 2017^11^. Compared to the estimated 0.02% case fatality rate for the global 2009 pandemic H1N1 virus^12^, A(H7N9) (including both high and low pathogenic strains) has an alarming 38% case fatality rate, which has resulted in 623 known deaths since its emergence in 2013^10,13,14^.

International travel can play a significant role in the spread of infectious diseases. For example, during the 2009 influenza H1N1 pandemic, countries receiving the highest volume of passengers (>1,400 in two months) from Mexico (the country of origin of the 2009 H1N1 virus), had a significantly higher risk of virus importation associated with travel to Mexico^15^. China (the country of origin of A(H7N4) and A(H7N9) viruses) is the third largest market of travelers for Singapore Changi Airport, which receives ~60,000 passengers from China per week. With Singapore’s close connection to China, and recent evidence suggesting the potential for airborne transmission of highly pathogenic avian influenza A(H7N9)^16^, it is prudent for Singapore to ramp up disease surveillance. The ability to quickly identify and robustly track respiratory threats is fundamentally important for an early emergency response.

To better understand the dynamics of microbes in cities in relation to human health, and to create healthier environments in areas where people frequently visit, researchers have begun studying the microbiome in and around public transportation vehicles^17,18^. An international project named MetaSUB^18^ was launched to map the microbiome of transit networks in the world’s largest cities. Over 50 cities, including Singapore, Boston, Shanghai, Paris, Stockholm, and São Paolo are participating in the project to improve city planning and public health. Although baseline metagenomic maps created from these studies are said to be useful for mitigating bioterrorism and infectious disease outbreaks, most of them focus largely on mapping surface-borne bacterial DNA^17^ and neglect to address the threat of weaponized or global catastrophic biological risk-level (GCBR-level) agents, both of which would likely be aerosolized or respiratory-borne RNA viruses^19^.

Bioaerosol studies have demonstrated that concentrations of airborne bacteria in underground subway systems tend to fluctuate seasonally and are linked to human presence and activities^20,21^. Such information demonstrates the possible role of public transportation in disease transmission, but again, only few studies have focused on the existence of airborne *viruses* in public transportation systems^22–25^. Mathematical models have been developed to assess the risk of airborne and droplet transmission of respiratory viruses in buses, airline cabins and cities^26–28^, and although these simulation data are useful, we have sparse field data to support them. In this study, we sought to monitor for respiratory viruses in Singapore’s Mass Rapid Transit (MRT) network, a likely area of virus incursion as well as a pathway for virus transmission. Specifically, we asked, could noninvasive bioaerosol sampling detect respiratory viruses in Singapore’s MRT network?

## Results

Using real-time polymerase chain reaction (PCR) and real-time reverse transcription PCR (RT-PCR), fourteen (16%) of the aerosol samples collected from the Singapore MRT tested positive for one or more respiratory viruses. Nine (64%) of the virus-positive aerosol samples were collected from the North East MRT Line (NEL; Purple) and five (36%) from the East West MRT Line (EWL; Green). Nine of the virus-positive aerosol samples (64%) tested positive for adenovirus, four (29%) tested positive for RSV-A, and one (7%) tested positive for influenza A virus. One sample tested positive for both adenovirus and RSV-A. One additional sample with a Ct value of 41 was suspected to be positive for influenza A virus. Aerosol samples did not test positive for any other target pathogens including influenza B viruses, enteroviruses, coronaviruses, and RSV-B.

Temperature, relative humidity (RH) and light intensity remained relatively consistent inside the Purple and Green MRT Lines throughout the study, with averages of 27 °C and 28 °C, 63% RH, and 710.4 and 730.7 lum/m², respectively (Table 1). There were no significant differences in these environmental conditions on sampling days yielding virus-positive aerosol samples.

**Table 1:** Virus-positive* aerosol samples collected from the Singapore MRT, 2017–2018.

Virus-positive Aerosol Samples	North East MRT Line (Purple)			East West MRT Line (Green)		
	Average			Average		
	Temperature (°C)	RH (%)	Light Intensity (lum/m²)	Temperature (°C)	RH (%)	Light Intensity (lum/m²)
	27	63	710.4	28	63	730.7
Influenza A virus	1 (>4 μm)			0** (>4 μm)		
Influenza B virus	0			0		
Adenovirus	6 (>4 μm; ≤4 μm)			3 (>4 μm)		
Enterovirus	0			0		
Coronavirus	0			0		
RSV-A	2 (>4 μm; ≤4 μm)			2 (>4 μm; ≤4 μm)		
RSV-B	0			0		
Total	9 (>4 μm; ≤4 μm)			5 (>4 μm; ≤4 μm)		

*Real-time RT-PCR/PCR Ct value < 40.

**One suspect-positive (Ct value = 41).

Adenovirus-positive droplets (>4 μm in aerodynamic diameter) and droplet nuclei (≤4 μm in aerodynamic diameter) were retrieved from six (67%) and four (44%) of the adenovirus-positive samples, respectively. In one instance, adenovirus-positive particles of both size ranges were retrieved from the same sample. RSV-A droplets and droplet nuclei were both retrieved from four (100%) of the RSV-A-positive samples. Only influenza A virus-positive droplets (not droplet nuclei) were retrieved from the influenza A virus-positive sample.

Adenovirus-positive samples were collected in January 2018, and February, March, May, June, September and October 2017. RSV-A-positive samples were collected in February 2017, and the influenza A virus-positive sample was collected in September 2017. The additional sample that was suspected to be positive for influenza A virus was collected in November 2017. See Table 2 for a monthly summary of PCR-positive aerosol samples.

**Table 2:** Monthly virus-positive* aerosol sample detections from the Singapore MRT, 2017–2018.

	Influenza A virus	Influenza B virus	Adenovirus	Enterovirus	Coronavirus	RSV-A	RSV-B
Jan 2017	—	—	—	—	—	—	—
Feb 2017	—	—	2	—	—	4	—
Mar 2017	—	—	2	—	—	—	—
Apr 2017	—	—	—	—	—	—	—
May 2017	—	—	1	—	—	—	—
June 2017	—	—	1	—	—	—	—
July 2017	—	—	—	—	—	—	—
Aug 2017	—	—	—	—	—	—	—
Sept 2017	1	—	1	—	—	—	—
Oct 2017	—	—	1	—	—	—	—
Nov 2017	— suspect**	—	—	—	—	—	—
Dec 2017	—	—	—	—	—	—	—
Jan 2018	—	—	1	—	—	—	—

*Real-time RT-PCR/PCR Ct value < 40.

**Real-time RT-PCR Ct value = 41.

Attempts to culture and subtype adenovirus and influenza A virus from PCR-positive aerosol samples were unsuccessful. Insufficient volumes of the original samples were leftover to attempt to culture RSV-A from PCR-positive aerosol samples.

## Discussion

To our knowledge, this is the first time molecular evidence for any infectious respiratory agent has been collected from Singapore’s MRT. Our results suggest that bioaerosol sampling might have a practical application for pathogen detection in public areas such as subway systems. Bioaerosol sampling in the field provides a noninvasive way to monitor and characterize the community of aerosolized respiratory viruses that regularly infect the public, as well as potentially detect or discover novel pathogens with pandemic potential, such as the influenza A(H7N9) virus. Additionally, a bioaerosol sampling system is advantageous as it does not require collecting individual samples from human subjects, nor informed consent.

Although this pilot study provides important qualitative data (i.e., molecular presence of respiratory viruses in the MRT), it is important to note that solely detecting viral DNA/RNA in the air does not determine that a virus is successfully transmitted through the air. Cultures in this study were negative and therefore the viruses detected may have been non-viable. However, studies have demonstrated that upon the first ~15 minutes of aerosolization, viruses tend to decay the most rapidly^29,30^, and therefore it is possible that the viability of the viruses captured in our study rapidly decreased during sample collection and transport. Moreover, temperature and RH have been widely understood to influence the viability of aerosolized viruses^31^. However, novel research has recently demonstrated that the viability of aerosolized influenza virus is independent of RH when the aerosol composition includes human bronchial epithelial (HBE) extracellular material (ECM)^32^, indicating that the influence of RH is more so on the rate of aerosol deposition after expulsion, which in turn, influences the overall concentration of viral aerosols in the environment. Aerosols tend to deposit onto surfaces more quickly at higher RH levels (>50%), suggesting that greater time spent in controlled environments with lower, more comfortable RH levels, could increase the risk of acquiring a respiratory virus via inhalation and respiration. Therefore, the average RH level (63%) on the Singapore MRT is not theoretically conducive to heavy airborne transmission of influenza viruses.

A likely contributor to the lack of data regarding airborne transmission of viruses is the difficulty of detecting respiratory pathogens in the air. Standard detection methods are designed to target viral nucleic acids which are subject to degradation from various environmental stressors (e.g., temperature, humidity, and UV light) as well as physical stress from aerosol sample collection methods, potentially deterring downstream detection. Therefore, high RH levels, in addition to sample degradation, could explain the low recovery of virus-positive aerosol samples in our study. Additionally, viral bioaerosols have notoriously low airborne concentrations, reducing the sensitivity of bioaerosol sampling. Some bioaerosol samplers, such as the NIOSH sampler used in this study, are compact and require a low flow rate which also may reduce sensitivity. Although the air pump flow rate and sample collection times used in our study have been demonstrated to efficiently capture aerosolized influenza virus and RSV RNA^33–35^, it is possible that these parameters are not optimal for capturing the other respiratory virus DNA/RNA targeted in our study. For example, it is possible that when compared to RNA viruses (e.g., influenza virus, RSV), adenovirus (a more durable DNA virus) can withstand a higher flow rate and/or longer sampling duration. Furthermore, although each of the assays used to detect viral DNA/RNA in our aerosol samples are highly sensitive, validated, singleplex real-time RT-PCR/PCR assays, specific detection limits (viral copies per volume of air) were not tested in our laboratory. Therefore, our DNA/RNA detections should not be mistaken as a true representation of the prevalence/densities of these viruses circulating in the air of the MRT, but rather a representation of the viruses our bioaerosol sampling technique is capable of detecting in this environment. To improve the sensitivity and specificity of bioaerosol sampling, more virus-specific controlled studies are needed to determine optimal sampling parameters for multiple respiratory viruses.

Airborne microbial concentrations have been demonstrated to fluctuate seasonally in underground subway systems^20^. However, the Singapore MRT is a controlled environment and therefore environmental conditions remained consistent throughout the study and no significant differences were recorded on days yielding virus-positive samples. Furthermore, unlike temperate climates with distinct seasons, Singapore has a tropical rainforest climate with high temperatures and humidity levels year-round, with little monthly variation. Therefore, Singapore experiences influenza outbreaks year-round, with two peak periods (April–June and September–December). Although we were able to stratify our pilot study data by month, our results are too limited to properly assess a correlation between virus-positive aerosols and Singapore’s peak influenza periods. However, it is notable that the influenza A virus-positive and suspect-positive aerosol samples were both detected during Singapore’s second peak influenza period in 2017. Additionally, RSV-A-positive aerosols were collected during weeks of increased polyclinic attendances for acute respiratory infections in Singapore^36^. Adenovirus-positive aerosols were collected during weeks of increased, average and decreased polyclinic attendances for acute respiratory infections^36^. A larger study yielding more aerosol samples over a longer duration would be needed to further characterize the relationship between bioaerosols in the MRT and community health data in Singapore.

Quickly identifying and robustly tracking respiratory threats are fundamental steps in an early emergency response to a pandemic. A bioaerosol sampling system is advantageous in this regard as it does not require the timely acquisition of ethical/IRB approvals and informed consent needed to collect individual samples from human subjects. If an outbreak is suspected or underway, bioaerosol samplers can be immediately deployed in high-risk areas, yielding results within a minimum of ~8 hours. Moreover, if bioaerosol samplers are proactively used to monitor high-risk areas, initial results turnaround times are nearly cut in half, as sample collection time is ~3–4 hours. Proactively monitoring for respiratory viruses also eliminates the risk of missing the time window of exposure to pandemic viruses in high-risk areas and would allow a more robust surveillance, which could be beneficial when the etiologic agent is known but the route of exposure is not fully understood.

Our bioaerosol sampling method also size-fractionates virus-laden particles, which can help measure the proportion of exposure to droplets versus droplet nuclei (i.e., inhalable versus respirable particles) in multiple environments and climates, which is important when assessing the type or severity of disease (e.g., an upper versus lower respiratory tract infection) that might follow. However, the full extent of exposure cannot be determined without measuring viral load. Our study falls short in that we did not quantify the viral load in our aerosol samples, making it difficult to compare our results with quantitative aerosol studies. In the future, we will implement a dilution series of positive controls into our real-time RT-PCR/PCR assay protocols to create a standard curve that can be used to accurately quantify the viral load in each aerosol sample.

When compared to a mobile/personal bioaerosol sampling method such as the one used in our study, a stationary sampling method is arguably a more practical approach to conducting disease surveillance.

**Figure 1:**
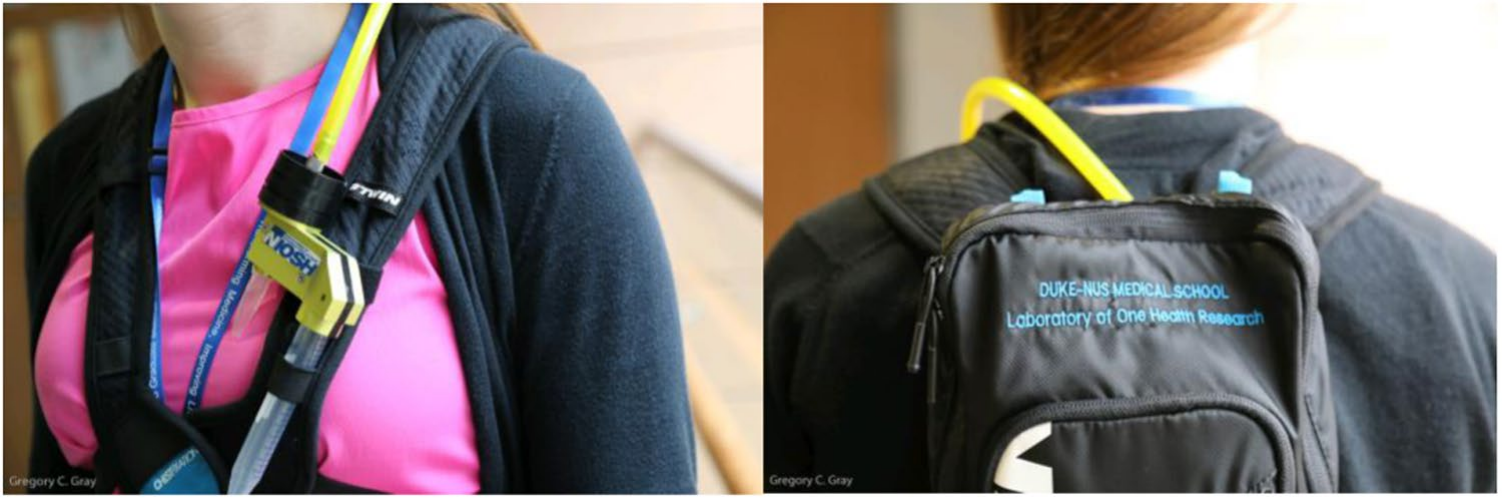
Aerosol sampler attached to a personal backpack carrying an SKC Airchek pump worn by researchers.

However, the use/installation of stationary samplers on the MRT would likely require permission from governmental authorities. Strapping the bioaerosol samplers to our bodies was the least invasive way for our team to recover molecular evidence of aerosolized respiratory viruses on the Singapore MRT. As this type of research is new to Singapore, the wellbeing of Singapore’s civilians was carefully considered and therefore, researchers wore their Duke-NUS employee badges while sampling, and our laboratory’s name was stitched onto the sampling backpacks (Fig. 1) for transparency. Similar to a study measuring the risk of exposure to aerosolized influenza virus among healthcare workers in an emergency department during influenza season^37^, strapping the samplers to personal backpacks allowed us to detect personal exposure to aerosolized viruses and provide insight into the efficacy of potential interventions (e.g., air/surface decontamination, and wearing face masks when infectious). Moreover, with evidence validating the notion that MRT riders are at risk of exposure to respiratory viruses, we hope to motivate scientists to conduct similar field studies to unveil the true risk of exposure while using public transportation, as data on this topic are scarce. Field studies may also inspire bioengineers and scientific instrument companies to design and test improved bioaerosol sampling such that it might be employed in a more widespread fashion to surveil for respiratory threats.

In conclusion, our study suggests that when combined with molecular diagnostics, aerosol sampling has promising potential to work as a noninvasive tool to monitor for respiratory pathogens in public areas. Additional studies are needed to assess a possible contribution of aerosol sampling to public health surveillance during periods of increased risk.

## Methods

### Bioaerosol sampling

From January 2017 through January 2018, National Institute for Occupational Safety and Health (NIOSH) BC 251 2-stage aerosol samplers were used to collect aerosol samples from the following Singapore Mass Rapid Transit (MRT) heavy rail lines: East West Line (EWL; green), and North East Line (NEL; purple). MRT lines were selected for their high capacity (~1,600 passengers per 6-car train), frequent use, and connection to high-traffic public areas. The EWL was specifically chosen for its connection to Changi Airport, and the NEL for its connection to downtown Singapore.

Aerosol samples were collected weekly, with occasional interruptions, during peak ridership (~267 passengers/car or 5 pax/m^2^) from 7:30–9:00 and 17:30–19:00 on Wednesdays. Sampling days varied occasionally due to public holidays and the researchers’ availability. Using a 2-foot (61 cm) long piece of ¼” (0.635 cm) Tygon tubing, NIOSH samplers were connected to AirChek® TOUCH Sample Pumps (SKC, Eighty Four, Pennsylvania) carried inside personal backpacks worn by the researchers. Both straps were tightened around the shoulders to secure the backpack against the researcher’s back. NIOSH samplers were securely fastened to the front of the backpacks (on or above the researcher’s heart) using Velcro (Fig. 1). For each separate MRT line (i.e., green and purple), the same assembled NIOSH sampler was used for both AM and PM sampling sessions. To ensure all researchers followed the same sampling protocol, each researcher sampled one of the middle train-cars and remained standing with intermittent walking throughout each sampling session. Between AM and PM sampling sessions, sampler inlets were covered with electrical tape and the assembled samplers were refrigerated. All of the material collected by one NIOSH sampler during 3 hours of sampling was counted as one aerosol sample.

Before aerosol sampling, air pumps were calibrated to sample at 3.5 L min^−1^ collecting a total of 630 L of air per MRT line each week. Calibration was performed by inserting the sampler into a calibration adaptor (designed for NIOSH samplers) attached to an SKC check-mate calibrator. NIOSH samplers were engineered to separate collected particles into three size fractions: >4 μm, 1–4 μm, and <1 μm in diameter^33^. Prior to molecular analysis, the particles collected in the 1–4 μm and <1 μm size fractions were combined (see sample processing below). Therefore, our study reports molecular results for two particle size fractions (droplets >4 μm and droplet nuclei ≤4 μm). If processed directly, the minimum time duration between the start of sample collection and reporting of assay results (for one, 3 hr aerosol sample) was 14 hours. However, most of the DNA/RNA extracted from the aerosol samples was stored at −80 °C and analyzed in monthly batches. NIOSH samplers were rinsed with deionized water and soaked in 70% ethanol between each sampling day and a swab sample of the sterilized NIOSH sampler interior was used as a negative control sample for each sampling day. Negative control swabs were transferred to tubes containing 1 mL of 0.5% BSA solution, vortexed for 15 seconds, and discarded. Negative control sample solutions were stored at −80 °C prior to further analyses.

### Environmental data collection

Ambient temperature, relative humidity (RH) and light intensity were logged during each sampling session using portable HOBO data loggers (Onset; Bourne, MA, USA) tied to the sampling backpacks worn by the researchers. Using Bluetooth Low Energy Technology, each HOBO data logger wirelessly transmitted data to the researcher’s mobile device using the HOBOmobile application. Once sampling was complete, researchers used the application to stop the data logger and convert the data file to an excel file before sending the data to a designated research assistant’s email address. Data were then recorded onto a master excel file.

### Sample processing

A UV-sterilized Biological Safety Cabinet Class II and sterile consumables and equipment were used to process samples. Using a filter-handling kit (225–8372; SKC), each polytetrafluoroethylene (PTFE) filter was removed from the NIOSH sampler cassettes and transferred to a 50 mL Falcon tube and vortexed while dry for 5 seconds. To minimize cross-contamination, forceps were sterilized with 70% ethanol in between each filter transfer. One milliliter of 0.5% BSA solution was then added to each 50 mL Falcon tube containing a filter and vortexed again for 15 seconds. One milliliter of 0.5% Bovine Serum Albumin (BSA) solution was added to each 1.5 mL conical tube from the NIOSH samplers and vortexed for 10 seconds. Using cryotube vials, the BSA solutions from the 50 mL Falcon tubes containing filters were pooled together with their respective 1.5 mL conical tube sample. Two milliliters of BSA solution were added to each 15 mL Falcon tube from the NIOSH samplers, vortexed for 15 seconds, and transferred to cryotube vials.

### Nucleic acid extraction and real-time RT-PCR/PCR

DNA/RNA was extracted from 0.5 mL of each aerosol sample and negative control sample solution using the QIAamp Viral RNA kit and QIAamp DNA Blood kit (Qiagen) following the manufacturer’s instructions. Using previously validated probe-based molecular assays^38–42^ adapted to the Duke-NUS Laboratory of One Health Research, extracted RNA was tested for the presence/absence of influenza A and B viruses, enteroviruses, coronaviruses, and RSV subtypes A and B using superscript III One-step real-time RT-PCR with Platinum Taq Polymerase. RSV-A and RSV-B assays were not adapted to our laboratory during the time of sampling and therefore were performed 3 months after the last aerosol sample was collected. Extracted DNA was tested for the presence/absence of adenoviruses by real-time PCR^43^ using a QuantiNova Probe PCR kit (Qiagen). Three negative control reactions (no template) were included in each real-time RT-PCR/PCR assay. A positive aerosol sample was one in which virus was detected in one or more of the NIOSH sampler stages, with a Ct value < 40. Stage 2 (1.5 mL tube) and stage 3 (filter) were combined to represent respirable particles (i.e., droplet nuclei ≤4 μm in diameter) compared with particles that are only inhalable (i.e., droplets >4 μm in diameter) collected in stage 1 (15 mL tube).

### Cell culture and DNA sequencing

Virus-positive samples were tested for viability using cell culture. First, 500 μL of each adenovirus-positive aerosol sample was inoculated into A549 cells (ATCC) with Dulbecco’s Modified Eagle Medium (DMEM) 2% (v/v) Fetal Bovine Serum (FBS) and incubated at 37 °C. MDCK cells were used to culture the influenza A virus-positive and suspect-positive aerosol samples. Inoculated shell vials were then observed for cytopathic effect 72 hours after inoculation, and daily for ten days afterwards. Observation of cytopathic effect was used to score positive or negative cultures. RSV-positive samples were not tested for viability as RSV assays were not performed until after all of the original aerosol sample material had been used for DNA/RNA extraction, leaving us with no original sample material to perform cell culture on RSV-positive samples. Extracted DNA from adenovirus-positive aerosol samples was typed using conventional PCR targeting the hexon (predicted amplicon size 764–896 bp) and fiber gene (predicted amplicon size according to species: A 1444–1537 bp; B 670–772 bp; C 1988–2000bp; D 1205–1221 bp; E 967 bp; F 541–586 bp).

## Declarations

The findings and conclusions in this report are those of the authors and do not necessarily represent the official position of the US Centers for Disease Control and Prevention.

## Data Availability

The datasets generated and/or analyzed during this study are available from the corresponding author on reasonable request.
